# Schisandrin B Ameliorates ICV-Infused Amyloid β Induced Oxidative Stress and Neuronal Dysfunction through Inhibiting RAGE/NF-κB/MAPK and Up-Regulating HSP/Beclin Expression

**DOI:** 10.1371/journal.pone.0142483

**Published:** 2015-11-10

**Authors:** Vijayasree V. Giridharan, Rajarajan A. Thandavarayan, Somasundaram Arumugam, Makoto Mizuno, Hiroyuki Nawa, Kenji Suzuki, Kam M. Ko, Prasanna Krishnamurthy, Kenichi Watanabe, Tetsuya Konishi

**Affiliations:** 1 J.K.K. Nattraja College of Pharmacy, Komarapalayam, Tamil Nadu, India; 2 Department of Cardiovascular Sciences, Houston Methodist Research Institute, Houston, Texas, United States of America; 3 Department of Clinical Pharmacology, Niigata University of Pharmacy & Applied Life Sciences (NUPALS), Niigata City, Japan; 4 Division of Neurobiology, Brain Research Institute, Niigata University, Niigata, Japan; 5 Department of Gastroenterology and Hepatology, Niigata University Graduate School of Medical and Dental Sciences, Niigata, Japan; 6 Section of Biochemistry and Cell biology, Hong Kong University of Science and Technology, Clear Water Bay, Hong Kong SAR, China; 7 Basic studies on second generation functional foods, NUPALS, NUPALS Liaison R/D promotion division, Niigata, Japan, Changchun University of Chinese Medicine, Changchun, RP China; Universidade de São Paulo, BRAZIL

## Abstract

Amyloid β (Aβ)-induced neurotoxicity is a major pathological mechanism of Alzheimer’s disease (AD). Our previous studies have demonstrated that schisandrin B (Sch B), an antioxidant lignan from *Schisandra chinensis*, could protect mouse brain against scopolamine- and cisplatin-induced neuronal dysfunction. In the present study, we examined the protective effect of Sch B against intracerebroventricular (ICV)-infused Aβ-induced neuronal dysfunction in rat cortex and explored the potential mechanism of its action. Our results showed that 26 days co-administration of Sch B significantly improved the behavioral performance of Aβ (1–40)-infused rats in step-through test. At the same time, Sch B attenuated Aβ-induced increases in oxidative and nitrosative stresses, inflammatory markers such as inducible nitric oxide syntheses, cyclooxygenase-2, interleukin-1β (IL-1β), IL-6, and tumor necrosis factor-α, and DNA damage. Several proteins such as receptor for advanced glycation end products (RAGE), nuclear factor-κB, mitogen-activated protein kinases, and apoptosis markers were over expressed in Aβ-infused rats but were significantly inhibited by Sch B treatment. Furthermore, Sch B negatively modulated the Aβ level with simultaneous up-regulation of HSP70 and beclin, autophagy markers in Aβ-infused rats. The aforementioned effects of Sch B suggest its protective role against Aβ-induced neurotoxicity through intervention in the negative cycle of RAGE-mediated Aβ accumulation during AD patho-physiology.

## Introduction

Extensive genetic and experimental evidences point to amyloid beta (Aβ) as the critical factor behind Alzheimer’s disease (AD) pathogenesis [1, 2]. This protein abnormally accumulates in the cortex and hippocampus of AD patient’s brain and has been shown to induce neuronal cell death directly [3, 4]. Previous studies have demonstrated that continuous intracerebroventricular (ICV) infusion of synthetic Aβ in rodent resulted in learning and memory deficits [5–7]. Evidence suggests that oxidative stress and neuronal inflammation, as well as neuronal apoptosis occur in the brains of chronic ICV Aβ-infused rats [8, 9].

Although great progress in understanding the etiology, pathology, and neurochemistry of AD has been achieved, the pathogenic mechanisms underlying this disorder are still unclear, besides oxidative stress is suggested as a key player [10, 11]. Aβ acts as a neurotoxin inducing oxidative stress directly and activating microglia indirectly, and thus certain specific cell-surface receptor has been postulated that guides Aβ onto target cells. “Receptor for advanced glycation end products” (RAGE) is one of such receptors that mediate Aβ effect on neurons and microglia. Increased expression of RAGE in AD brain is implicated in the pathogenesis of neuronal dysfunction and death [10]. It is also reported that in many cell types, AGE binding to the receptor induces patho-physiological cascades linked to the downstream activation of NF-κB and other signaling pathways, which subsequently lead to reactive oxygen species generation and certain pro-inflammatory responses [12]. The possibility of interfering with this detrimental cycle by pharmacologically inhibiting RAGE expression and reactive gliosis has been proposed as one of the targets for developing drugs to reduce neuronal damage and consequently slow the progress of AD.

Schisandrin B (Sch B) is a dibenzocyclooctadiene derivative isolated from the fruit of *Schisandra chinensis* (FS), a traditional Chinese herb commonly used as an astringent, and has been clinically used for the treatment of viral and chemical hepatitis and myocardial disorders [13, 14]. Recently, the neuroprotective potential of Sch B has been studied by us and also by other group in several *in vivo* and *in vitro* model systems such as against scopolamine induced amnesia, Aβ-induced neurotoxicity and cisplatin-induced oxidative stress, genotoxicity, and neurotoxicity [15, 16]. Therefore, the neuroprotective function of Sch B might be beneficial for preventing AD pathogenesis. In the present study, we examined the effect of Sch B against ICV-infused Aβ-induced amyloidogenesis, by focusing on the potential of Sch B as an antioxidant and anti-inflammatory agent, and an inhibitor of RAGE/NF-κB/MAPK expression and modulator of autophagy marker in preventing Aβ-induced neuronal dysfunction.

## Materials and Methods

### Animals

Male Sprague Dawley rats (from SLC, Japan) weighing 240–300 g were housed in a controlled environment at 25±2°C with alternating 12 hour light and dark cycles. All rats were acclimatized in our animal facility for at least 1 week before the experiments were conducted. Rats were maintained with free access to water and chow throughout the period of study, and animals were treated in accordance with the Guidelines for Animal Experimentation of our institute. All animal protocols used in this study were approved by the Institutional Review Board at Niigata University of Pharmacy and Applied Life Sciences.

### Drugs and chemicals

Aβ (1–40) peptide, acetylthiocholine iodide (AChI), and 5,5'dithiobis(2-nitrobenzoic acid) (DTNB) were purchased from Sigma-Aldrich USA. All other chemicals used in the study were of analytical grade. Solutions of the drug and chemicals were freshly prepared before use.

### Surgery and experimental design

**Fig 1:**
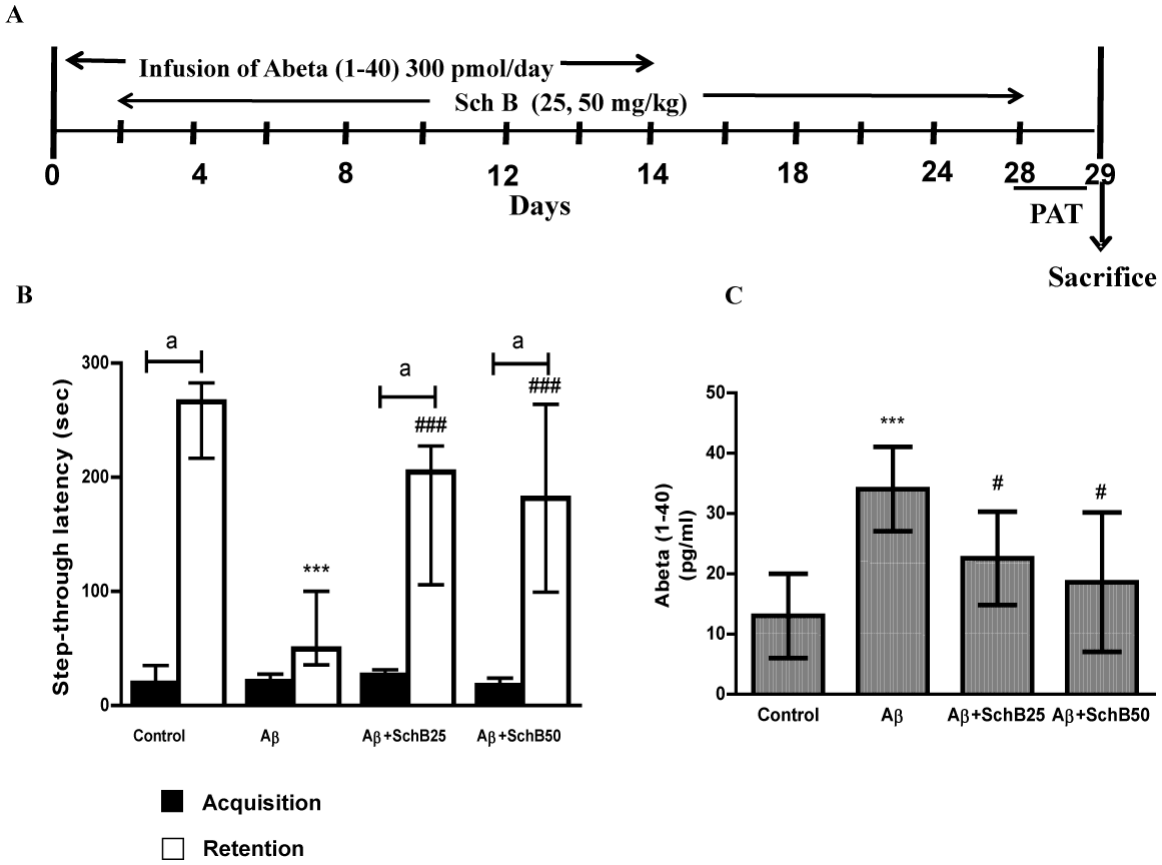
**(A) Experimental schedule (B) Effect of Sch B (25 and 50 mg/kg) on Aβ level using ELISA method.** Aβ levels were measured in cortex homogenate of Aβ/vehicle-infused rats (n = 5). **(C) Effect of Sch B (25 and 50 mg/kg) on step-through latency of single-trial PAT.** The acquisition and retention trials were carried out on days 28 and 29, respectively (n = 6). Data are presented as the median±inter quartile. *** *P* < 0.001, statistically different from control group. ^###^ *P* < 0.001, ^#^ *P* < 0.05 statistically different from Aβ-infused rats, ^a^ *P* < 0.05 statistically different from the test session with respective training session.

Aβ (1–40) peptide was dissolved in 35% acetonitrile/0.1% trifluoroacetic acid. Rats were anesthetized with pentobarbital (50 mg/kg i.p.). The skull was exposed and drilled relative to the bregma (A, 0.8; L, 1.4; V, 4.5) according to the atlas of Paxinos and Watson using a stereotaxic frame (Narishige, Tokyo, Japan) [17]. A mini-osmotic pump (Alzet 2002; Durect Co., Cupertino, CA, USA), loaded with Aβ (1–40) peptide (300 pmol/day), was quickly implanted into the backs of the rats. Control animals were infused with vehicle (35% acetonitrile/0.1% trifluoroacetic acid) alone. The experimental schedule is shown in Fig 1A. Aβ (1–40) or vehicle infusion began on day 0 and continued till day 14 [7, 8, 18]. Animals were divided into 4 groups (n = 8) and the groups were 1.Sham control (vehicle infused) 2. Aβ-infused 3. Aβ-infusion+Sch B 25 4. Aβ-infusion+Sch B 50. Sch B was dissolved in olive oil and orally administered via tubing to the stomach at a concentration of 25 or 50 mg/kg from day 3 to day 28. Control animals were orally administered olive oil. The behavioral study was performed on days 28 and 29. The animals were sacrificed on day 29 and brain cortex was preserved for biochemical and histo-chemical evaluations.

#### Brain tissue preparation

On day 29, the rats were decapitated and the cerebral cortex was quickly removed, blotted gently with filter paper to remove blood and extraneous tissues, and frozen with liquid nitrogen to store at -80°C until use.

### Behavioral study

#### Passive avoidance task (PAT)

Training for and testing of passive avoidance performance were carried out in two identical light and dark square boxes [19]. On day 24, the rats were initially placed in the light chamber and 10 seconds later the door between the compartments was opened. When rats entered the dark compartment, the door was closed and an electrical foot shock (0.1 mA/10 g body weight) for a period of 2 seconds was delivered through stainless steel rods (one training trial). The last shots of Sch B were given 1 hour before the training trial. Twenty-four hours after the training trial, the rats were again placed in the light compartment. The step-through latency to enter the dark compartment was measured [20]. If the rat did not enter the dark compartment within 300 seconds, the experiment was stopped.

### Biochemical analysis

For biochemical analysis, the cortex region was weighed and homogenized (10% w/v) in ice-cold sodium phosphate buffer (30 mM, pH 7.0).

### Measurement of Aβ (1–40) level

The levels of Aβ peptide (1–40) were analyzed with a colorimetric sandwich ELISA kit according to the manufacturer’s instructions (IBL Co., Ltd., Japan).

### Assays of acetylcholinesterase (AChE), acetylcholine (ACh), and nitrite and oxygen radical absorbing capacity (ORAC)

AChE activity was determined according to the colorimetric assay of Ellman, as previously described [21]. ACh was determined by the method of Hestrin [22]. The accumulation of nitrite, an indicator of the production of nitric oxide, was determined using Griess reagent as described by Green et al. [23]. ORAC was measured using disodium fluorescein as a fluorescence probe, and 2,2-azobis(2-amidinopropane) dihydrochloride (AAPH) as a peroxyl radical generator, which is relevant to biological systems because the peroxyl radical is the most abundant free radical [24].

### Lipid peroxidation and antioxidant assays

The quantitative measurement of malondialdehyde (MDA), an end product of lipid peroxidation, in rat cortex was performed according to the method of Ohkawa et al. [25]. Reduced glutathione (GSH) was determined by the Ellman method [26]. The superoxide dismutase activity (SOD) was determined using the water-soluble tetrazolium method [27].

### *In situ* detection of superoxide production in cortex

To evaluate *in situ* superoxide production, unfixed frozen cross sections of the specimens were stained with dihydroethidium (DHE; Molecular Probes, Eugene, OR, USA) according to a previously validated method [28].

### Single-cell gel electrophoresis method (comet assay)

We adopted a standard protocol for the comet assay preparation and analysis [29–31]. About 20 μl of cortex suspension was supplemented with 0.8% low-melting-point agarose solution prepared in 0.9% saline. Then, this mixture was poured onto fully frosted microscope slides and subjected to lysis. Electrophoresis was carried out for 30 minutes at 25 V and the slides were washed gently in a neutralizing buffer (0.4 M Tris-HCl, pH 7.5) and stored at 4°C until observation. The slides were stained with SYBR green II, and at least 50 cells were captured per slide at ×200 magnification using a fluorescence microscope (Olympus (BH2-RFCA), Japan). The comet images were analyzed by the digital imaging software CASP.

### Western immunoblotting

The cortex obtained from different rat groups was homogenized with lysis buffer. Protein concentrations in these homogenized samples were measured by the bicinchoninic acid method. For Western blots, 30 μg of protein was separated by SDS-PAGE and identified with the following antibodies to quantify the levels of protein: p53, P-p53, anti-glyceraldehyde-3-phosphate dehydrogenase (GAPDH), anti-tumor necrosis factor-α (TNF-α), phospho-nuclear factor kappa B (NF-κB), p-Iκ-α, p38 mitogen-activated protein kinase (p38 MAPK), phospho-p38 MAPK, receptor for advanced glycation end product (RAGE), heat shock protein-70 (HSP-70), BECN-1 (beclin-1) (Santa Cruz Biotechnology, Santa Cruz, CA, USA), β-tubulin, p-ERK, ERK (Cell Signaling Technologies, NJ, USA). We used 10% sodium dodecyl sulfate-PAGE for separating proteins (Bio-Rad, CA, USA), and separated proteins were electrophoretically transferred to PVDF membranes. Membranes were blocked with 4% skimmed milk in Tris-buffered saline (TBS)T. The primary antibodies were (1:1000) incubated with the membrane for 16 hours at 4°C. Secondary antibodies (1:10,000) conjugated with horseradish peroxidase (Santa Cruz Biotechnology, Santa Cruz, CA, USA) were incubated for 1 hour at room temperature. The target proteins were visualized using an ECL reaction kit (Amersham, NJ, USA) and chemiluminescence film (Amersham). Films were scanned, and band densities were quantified by densitometric analysis using Scion Image program (GT-X700, Epson, Tokyo, Japan).

### Histological examination

On day 29, after behavioral experiments, the rats were anesthetized and transcardially perfused with PBS followed by paraformaldehyde, and then the coronal sections were fixed with 4% paraformaldehyde and embedded in paraffin. The paraffin-embedded sections were cut to5 μm sections using a microtome.

### Immunohistochemistry

Paraffin-embedded brain sections were cut at 5 μm and affixed to slides to ensure adhesion. After deparaffinization and hydration, the slides were washed in TBS (10 mM/l Tris HCl, 0.85% NaCl, pH 7.5) containing 0.1% bovine serum albumin (BSA). Endogenous peroxidase activity was quenched by incubating the slides in methanol and 0.6% H_2_O_2_ in methanol. The primary antibodies, inducible-nitric oxide synthase (iNOS; 1:200) and cyclo-oxygenase-2 (COX-2; 1:200) (Santa Cruz Biotechnology, Santa Cruz, CA, USA) were left overnight at 4°C. The slides were washed in TBS and then horseradish peroxidase (HRP)-conjugated secondary antibody (1:500) (Santa Cruz Biotechnology, Santa Cruz, CA, USA) was added and the slides were further incubated at room temperature for 45 minutes. The slides were washed in TBS and incubated with diaminobenzidine tetrahydrochloride as the substrate, counterstained with hematoxylin, and observed under a light microscope.

### Immunofluorescence

For immunofluorescence, tissues were fixed in 10% buffered formaldehyde solution and embedded in paraffin. Sections underwent microwave antigen retrieval, were blocked with 10% serum in phosphate-buffered saline, and were incubated with anti-caspase-3 (1:200) (Santa Cruz Biotechnology, Santa Cruz, CA, USA). Binding sites of the primary antibody was revealed using a fluorescein isothiocyanate-conjugated secondary antibody (1:500) (Sigma–Aldrich, St. Louis, MO, USA). Samples were visualized with a fluorescence microscope at 400× magnification (CIA-102; Olympus).

### Gene expression analysis by real-time reverse transcription polymerase chain reaction (RT-PCR)

Cortex was preserved by immersion in RNA-later solution (Ambion Inc., Austin, TX) immediately after sampling. The extraction of total RNA was performed after homogenization using Ultra TurraxT8 (IKA Labortechnik, Staufen, Germany) in TRIzol reagent (Invitrogen Corp., Carlsbad, CA) in accordance with the standard protocol. Synthesis of cDNA was performed by reverse transcription using total RNA (2 μg) as a template (Super Script II; Invitrogen Corporation, Carlsbad, CA). Gene expression analysis was performed by RT-PCR (Smart Cycler; Cepheid, Sunnyvale, CA) using cDNA synthesized from the cortex specimen. Primers, namely, for interleukin (IL)-1β, TNF-α, IL-6, and hemoxygenase-1 (HO-1), were used. Real-time RT-PCR by monitoring with TaqMan probe (TaqMan Gene expression assays; Applied Biosystems, Foster City, CA) was performed in accordance with the following protocol: 600 seconds at 95°C, followed by thermal cycles of 15 seconds at 95°C and 60 seconds at 60°C for extension. Relative standard curves produced using several 10-fold dilutions (1:10:100:1000:10,000:100,000) of cDNA from the cortex were used for linear regression analysis of other samples. Results were normalized to GAPDH mRNA as an internal control and are thus shown as relative mRNA levels.

### Analysis of neuronal apoptosis by terminal transferase-mediated dUTP nick-end labeling (TUNEL) assays

TUNEL assays were performed as specified in the instructions for the *in situ* apoptosis detection kit (Takara Bio Inc., Shiga, Japan). Digital photomicrographs were obtained by using a color image analyzer (CAI-102, Olympus) and 25 random fields from each cortex were chosen and the number of TUNEL-positive nuclei was quantified in a blinded manner.

### Statistics

The heteroscedasticity of data from passive avoidance task and the use of the 300-second cut off time in test sessions required the use of nonparametric statistics. The difference between the acquisition and retention within each group were analyzed using Wilcoxon matched pair test and the results were expressed as median ± inter quartile [32]. Besides, to verify the difference between groups, one way analysis of variance (ANOVA) followed by Tukey’s post hoc was performed. All other data were analyzed by one way ANOVA followed by Tukey’s post hoc or by t test when appropriate. *P* < 0.05 were considered statistically significant. Graph pad Prism version 5.0 was used for statistical analysis. All the results are expressed as mean ± standard deviation (SD).

## Results

### Effect of Sch B on Aβ level in the cortex of Aβ-infused rats

As the result of Aβ perfusion, there was a significant increase (34±7 pg/ml) in the level of Aβ in the cortex compared with that of the vehicle-infused (13±6 pg/ml) rats. However, in the rats treated with Sch B negatively modulated the elevated level of Aβ and the levels decreased to 22±7 pg/ml and18±11 pg/ml in Sch B 25 and 50 groups, respectively (Fig 1B).

### Effect of Sch B on memory impairment induced by Aβ infusion

During the training (acquisition) in PAT, we observed no significant difference between any groups. Animals from different groups entered the dark chamber in short time. However, in test (retention) session that performed 24 hour after training, we observed significant difference with in the groups. Control group showed a significant increase in latency to enter the dark chamber (266 sec; *P* < 0.05), as compared to its training suggests their memory retrieval. Similarly, the latency to enter the dark chamber was significantly improved in Sch B 25 (204.5 sec; *P* < 0.05) and Sch B 50 (181.5 sec; *P* < 0.05) as compared to respective training session, suggesting their ability to remember the task. However, Aβ–alone infused group presented a latency time similar to the time in the training session (49.5 sec) suggesting that they did not retained the information. In addition, one way ANOVA followed by Tukey’s post hoc test revealed the difference between the groups. We observed that Aβ infusion significantly (249 Vs 60 seconds) decreased the step-through latency in the retention trial, but Sch B both at 25 and 50 mg/Kg significantly prevented the Aβ-induced cognitive deficits in the PAT, although clear dose dependence was not observed (Sch B 25:178±60 seconds and Sch B 50:181±82 seconds, respectively)(Fig 1C).

### Effect of Sch B on cholinergic modulators (AChE, ACh)

**Fig 2:**
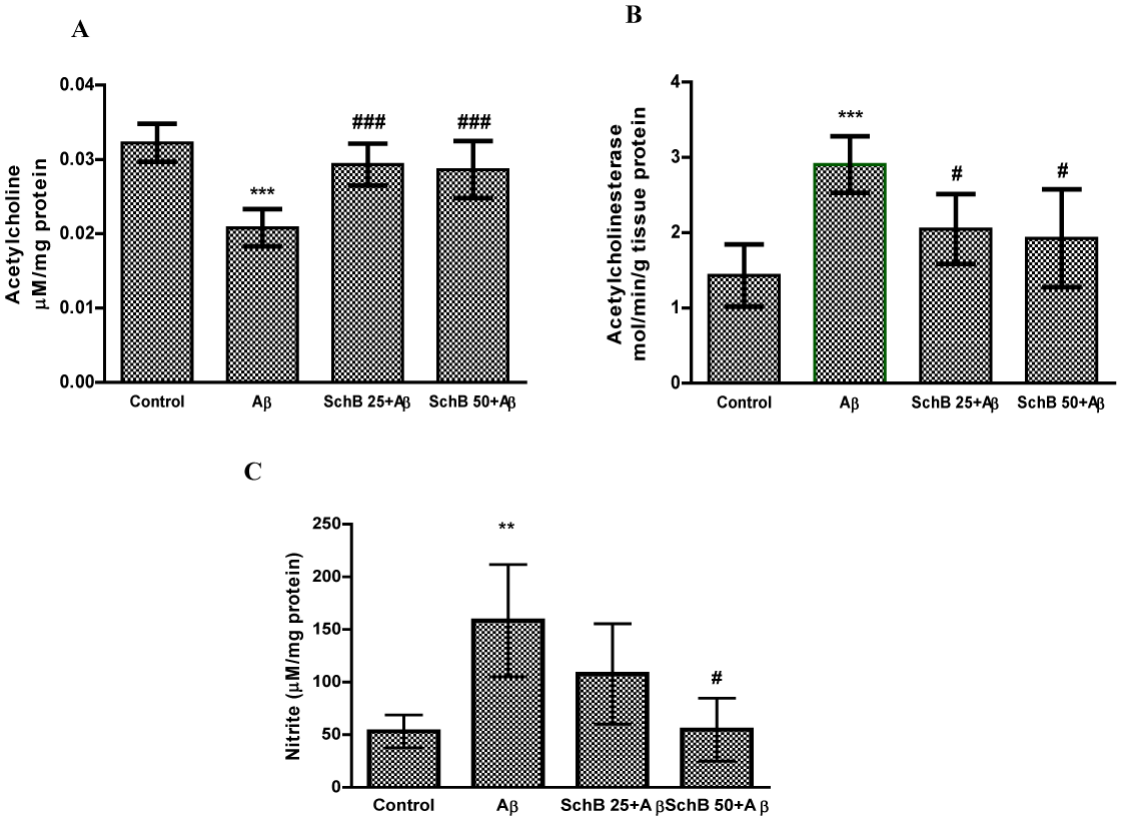
Effect of Sch B on (A) acetylcholinesterase (AChE) (B) acetylcholine (ACh), and (C) nitrite levels in Aβ/vehicle infused rats. Data are represented as the mean ± SD (n = 5–6). *** *P* < 0.001, **P < 0.01, statistically different from control group. ^###^ *P* < 0.001, ^#^ *P* < 0.05 statistically different from Aβ-infused rats.

In AD patients, the ACh level in the brain is significantly reduced due to the loss of neurons including basal forebrain cholinergic neurons and also the activation of the hydrolyzing enzyme AChE [33]. Therefore AChE inhibitor is the target for treating AD to prevent ACh decomposition, thereby improve cognitive and behavioral symptoms [34]. It was found in the present study that Aβ infusion led to an increase in AChE (2.9±0.3 mol/min/g) activity as compared to the vehicle infused (1.4±0.4 mol/min/g) group. Consistently with this increase of AChE activity, the ACh (0.02±0.002 μM/mg) level in cortex was significantly decreased in the Aβ infused group compared with those of the vehicle (0.03±0.002 μM/mg) group. Administration of Sch B significantly suppressed the increase of AChE (*P* < 0.05) activity and improved the ACh (*P* < 0.001) level back to the vehicle control both in Sch B 25 and 50 groups (Fig 2A and 2B).

### Effect of Sch B on NO release in Aβ-infused rats

The release of NO was evaluated by measuring its stable metabolite (NO_2_^-^) using cortex homogenate. The NO_2_^-^ level in the cortex was significantly increased (158±53 μM/mg) by Aβ infusion in comparison with control (53±15 μM/mg) group. However, Sch B inhibited the NO production, significantly, at the dose of 50 mg/kg (54±29 μM/mg) (Fig 2C).

### Effect of Sch B on ORAC value and oxidative stress markers

The ORAC assay has been widely accepted as a tool for assessing the antioxidant potential of foods and natural products as well as plasma and tissues [35]. Table 1 show the antioxidant capacity of Sch B assessed by ORAC value, where one ORAC unit equals the fluorescence decay inhibited by 1 μM trolox as an antioxidant reference. The data clearly demonstrated that Sch B increased the ORAC value in the plasma of Aβ infused rat both at Sch B 25 (*P* < 0.01) and Sch B 50 (*P* < 0.001) and thus protected against damages induced by oxygen free radicals produced by Aβ. The Aβ induced oxidative stress was also evident as lipid peroxidation was accelerated in the brain of Aβ-infused (*P* < 0.01) rats compared to vehicle control but was significantly inhibited in Sch B 25 and Sch B 50 (*P* < 0.01) treated groups. Consistently, antioxidant GSH level in the brain was decreased in Aβ-treated rats (*P* < 0.05) but this decrease in GSH was finely prevented by Sch B 25 and Sch B 50 (*P* < 0.01) treatment (Table 1). We further examined the modulation effect of Sch B on antioxidant enzyme SOD by biochemical method. The SOD level was decreased (*P* < 0.001) in Aβ-infused rats but Sch B treatment prevented the decrease both in Sch B 25 (*P* < 0.01) and Sch B 50 (*P* < 0.001) groups, respectively (Table 1).

**Table 1:** Effect of Sch B on oxygen radical absorbing capacity (ORAC), malondialdehyde (MDA), glutathione (GSH), and superoxide dismutase (SOD) levels in Aβ/vehicle infused rats.

Groups	ORAC (Trolox equivalent, μM)	MDA (μM/mg protein)	GSH (μM/mg protein)	SOD (U/mg protein)
Control	17293±1654	0.651±0.265	2.435±0.955	8.167±0.763
Aβ	8372±4259**	1.604±0.455**	0.625±0.871*	2.072±1.352***
Aβ+SchB25	15800±1544^##^	0.810±0.230^##^	2.392±0.980^#^	5.342±1.944^##^
Aβ+SchB50	18238±1575^###^	0.546±0.292^##^	2.534±1.027^#^	6.729±0.870^###^

Data are represented as the mean ± SD (n = 5).

*** *P* < 0.001,

**P < 0.01,

* *P* < 0.05 statistically different from control group.

^###^ *P* < 0.001,

^##^ *P* < 0.01,

^#^ *P* < 0.05 statistically different from Aβ-infused rats.

### Effect of Sch B on NF-κB, p38/ERK and RAGE in Aβ-infused rats

The oxidative stress is associated with activation of cellular signaling pathways such as MAPK and NF-κB, and also production of pro-inflammatory cytokines, moreover, these play critical role in Aβinduced neuronal cell death.

**Fig 3:**
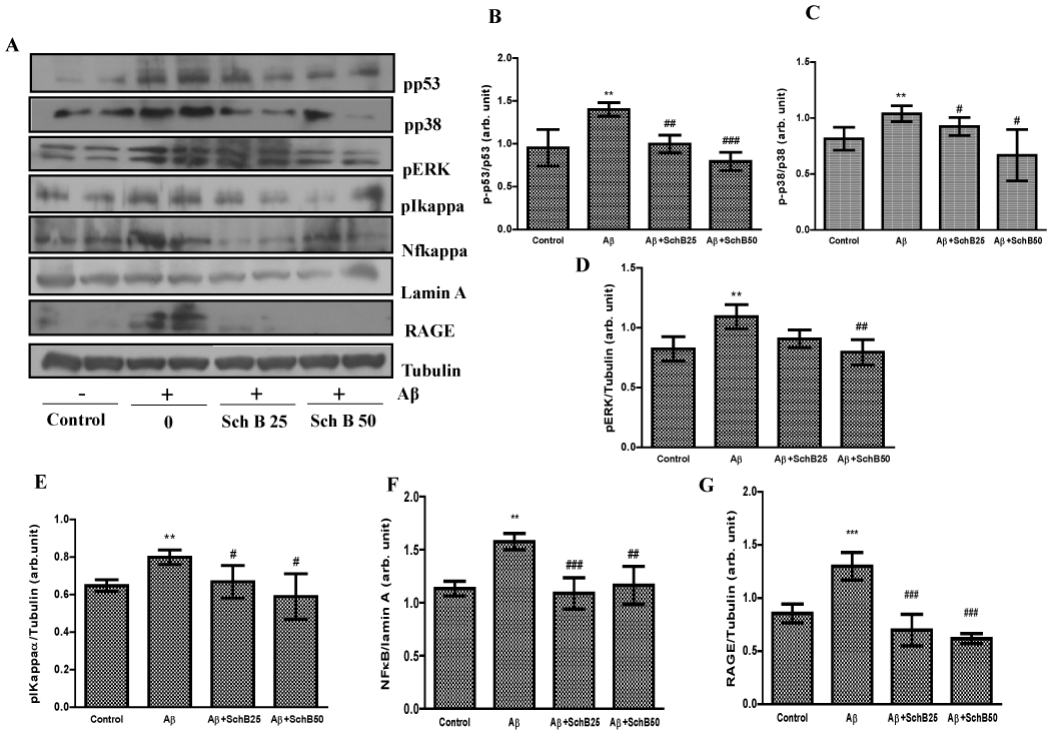
Sch B (25 and 50 mg/kg) administration inhibits the activation of the MAPK/NF-κB/RAGE axis in the cerebral cortex of Aβ-infused rats. (A) Representative immunoblots for p-p53, p-p38, p-ERK, p-Iκα, NFκB, and RAGE expression levels. The expression levels are expressed as ratio of (B) p-ERK/tubulin, (C) p-p38/p38, (D) p-p53/p53, (E) p-Iκα/tubulin, (F) NF-κB/lamin and (G) RAGE/tubulin. The results are given as the mean ± SD (n = 4). *** *P* < 0.001, **P < 0.01, compared with control group, and ^###^ *P* < 0.001, ^##^ *P* < 0.01, ^#^ *P* < 0.05, significantly different from Aβ-infused rats.

We first examined the effects of Sch B on MAPK activation by Western blotting (Fig 3A–3C). As was reported elsewhere both phosphorylated ERK and p38 were over-expressed *(P* < 0.01) in Aβ infused rats [36, 37], but the expressions were significantly attenuated by Sch B treatment.

The effect of Sch B on NF-κB was studied also by Western blotting as described elsewhere [38]. NF-κB activation was also significantly enhanced in the Aβ infused *(P* < 0.01) rats but Sch B treatment effectively attenuated both the Aβ-induced NF-κB (Sch B 50 mg/kg *(P* < 0.01) and p-Iκα (Sch B 50 mg/kg *(P* < 0.05) indicating Sch B modulated NF-κB activity through IκB phosphorylation (Fig 3E and 3F).

Since Sch B attenuated Aβ level in the brain (Fig 1B), the effect of Sch B on RAGE was further examined by Westernblotting. RAGE is a multi-ligand receptor in the immunoglobulin family, expressed in tissues with ongoing glycol-oxidation and leads the tissues to damage. It is also associated with deposit of Aβ in the brain [39]. RAGE expression is also observed in the activated microglial cells and astrocytes in neurodegenerative disease [40, 41]. Similarly in the present study, Aβ infusion significantly (*P* < 0.001) increased the number of RAGE-positive cells in cerebral cortex when compared with that in vehicle control. However, Sch B co-administration to Aβ-infused rats significantly (*P* < 0.001) reduced the number of RAGE-positive cells as shown in Fig 3G.

### Effect of Sch B on oxidative DNA damage and autophagy expression

**Fig 4:**
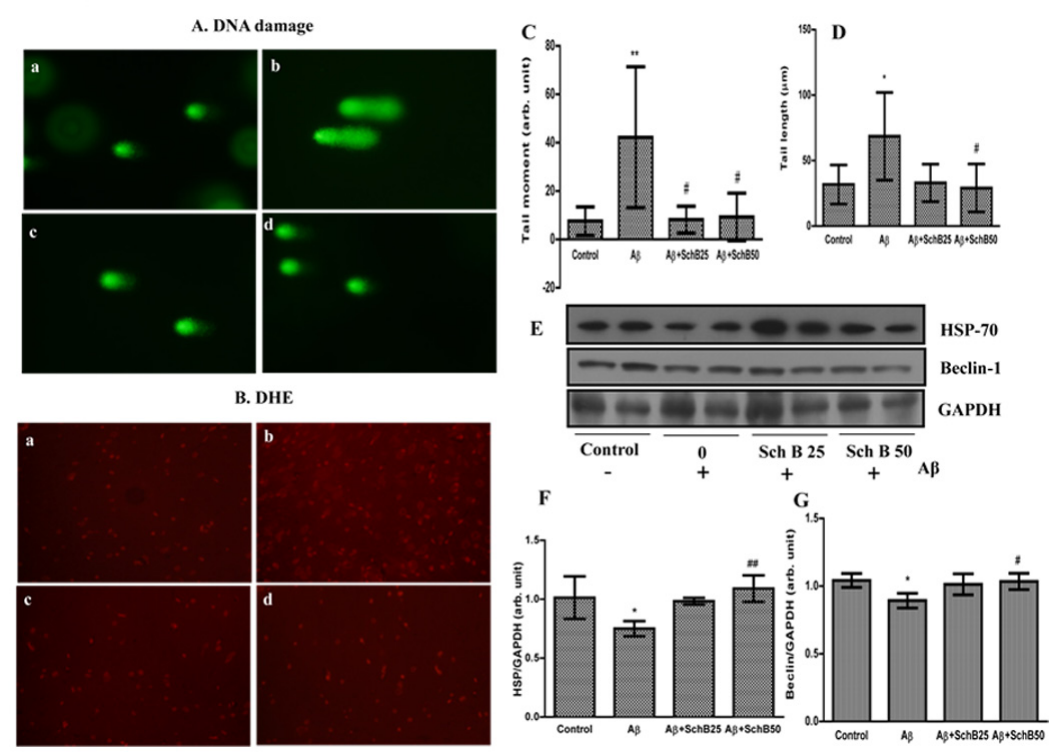
Sch B (25 and 50 mg/kg) administration reduced the DNA damage, superoxide production and moderately increases the HSP-70 and beclin-1 expression in the cerebral cortex of the Aβ-infused rats. (a) Control (b) Aβ-infused (c) Aβ+SchB25 (d) Aβ+SchB50. (A) Representative photomicrograph of DNA damage detected by single-cell gel electrophoresis assay (comet assay). (B) Representative photomicrograph of *in situ* superoxide production (bright area) using DHE staining in cortex. (C) Tail moment (D) Tail length analyzed by digital imaging Casp software (http://casp.sourceforge.net/). (E) Representative immunoblots for HSP-70, and beclin-1 expression levels. The expression levels are expressed as ratio of (F) HSP-70/GAPDH, and (G) beclin-1/GAPDH. The results are given as the mean ± SD (n = 4). * *P* < 0.05, compared with control group, and ^##^ *P* < 0.01, ^#^ *P* < 0.05, significantly different from Aβ-infused rats.

Oxidative DNA damage was evaluated by performing the alkaline comet assay. The DNA damage in the brain cortex assessed by tail moment was significantly (*P* < 0.01) increased in the Aβ infused rats but was significantly (*P* < 0.05) less in Sch B-treated rats (Fig 4A). The marked increase (68±33 Vs 31±14 μm) of tail length in the Aβ infused rats was significantly attenuated by the treatment with Sch B 25 (33±14 μm) and Sch B 50 (29±18 μm) suggesting the protective effect of Sch B against Aβ-induced DNA damage (Fig 4C and 4D).

Superoxide radical production in the cortex was also studied histochemically using a superoxide radical specific fluorescent probe DHE. DHE reacts with superoxide to produce ethidium that in turn intercalates into DNA to generate red fluorescence. The intracellular red fluorescence was significantly enhanced after Aβ infusion as shown in Fig 4B. The Aβ-induced fluorescence level was low in the Sch B treatment group comparable to that of vehicle control, indicating an overall reduction of oxidative stress by Sch B.

In addition to the RAGE over expression, scavenging of Aβ the another mechanism involved in the decreased accumulation of Aβ by Sch B, and thus the effects of Sch B on HSP and beclin 1 were studied. The results showed HSP70 expression was considerably increased in Sch B 50 (*P* < 0.01) treated rats when measured by Western blotting (Fig 4E and 4F). Beclin 1 expression was also moderately (*P* < 0.05) increased in Sch B administration at 50 mg/kg (Fig 4E and 4G).

### Effect of Sch B on inflammatory markers

**Fig 5:**
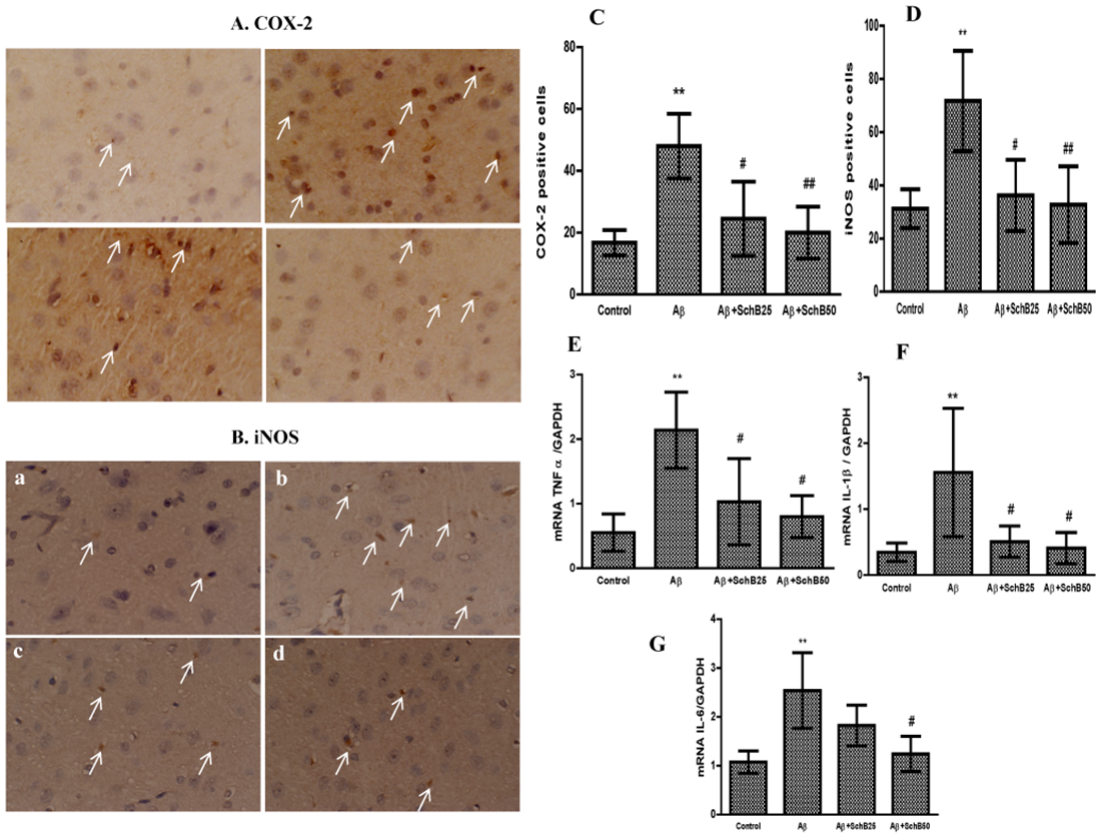
Inhibitory effect of Sch B (25 and 50 mg/kg) treatment on inflammatory markers. (a) Control (b) Aβ-infused (c) Aβ+SchB25 (d) Aβ+SchB50. Representative immunohistochemical detection of (A) COX-2 (B) iNOS-positive cells. The figures show quantification of (C) COX-2 (D) iNOS-positive cells. From each rat 5–6 coronal brain sections were selected and about 15 fields of the cerebral cortex were analyzed. Scale bar = 5μm (E) TNF-α, (F) IL-β, and (G) IL-6, mRNA expression in rat cerebral cortex quantified by densitometry. The results are given as the mean ± SD (n = 4). ** *P* < 0.01, compared with control group, and ^##^ *P* < 0.01, ^#^ *P* < 0.05, significantly different from Aβ-infused rats.

We further examined the expression of inflammatory markers, including TNF-α, IL-1β, IL-6, tissue growth factor (TGF), and interferon-γ (IFNγ) in the cerebral cortex of each group by RT-PCR. The inflammatory enzymes COX-2 and iNOS expression were measured by immunohistochemical method. We found a significant increase (*P* < 0.01) in the number of inflammatory marker enzymes COX-2 and iNOS immunopositive cells in Aβ-infused rats. In the Sch B 25 (*P* < 0.05) Sch B 50 (*P* < 0.01) treated groups, these inflammatory marker enzymes were significantly attenuated in the cortex of the Aβ-infused group (Fig 5A–5D). Further, Aβ-infused rats showed significant (*P* < 0.01) increases of TNF-α, IL-1β, and IL-6 mRNA expressions, but the treatment with Sch B 25 (*P* < 0.05) Sch B 50 (*P* < 0.05) was found to significantly attenuate these inflammatory markers in the cortex (Fig 5E–5G). However, no significant difference was found in terms of TGF or IFNγ mRNA expression after treatment with Sch B (data not shown).

### Effect of Sch B on apoptotic neuronal cell death in Aβ-infused rats

**Fig 6:**
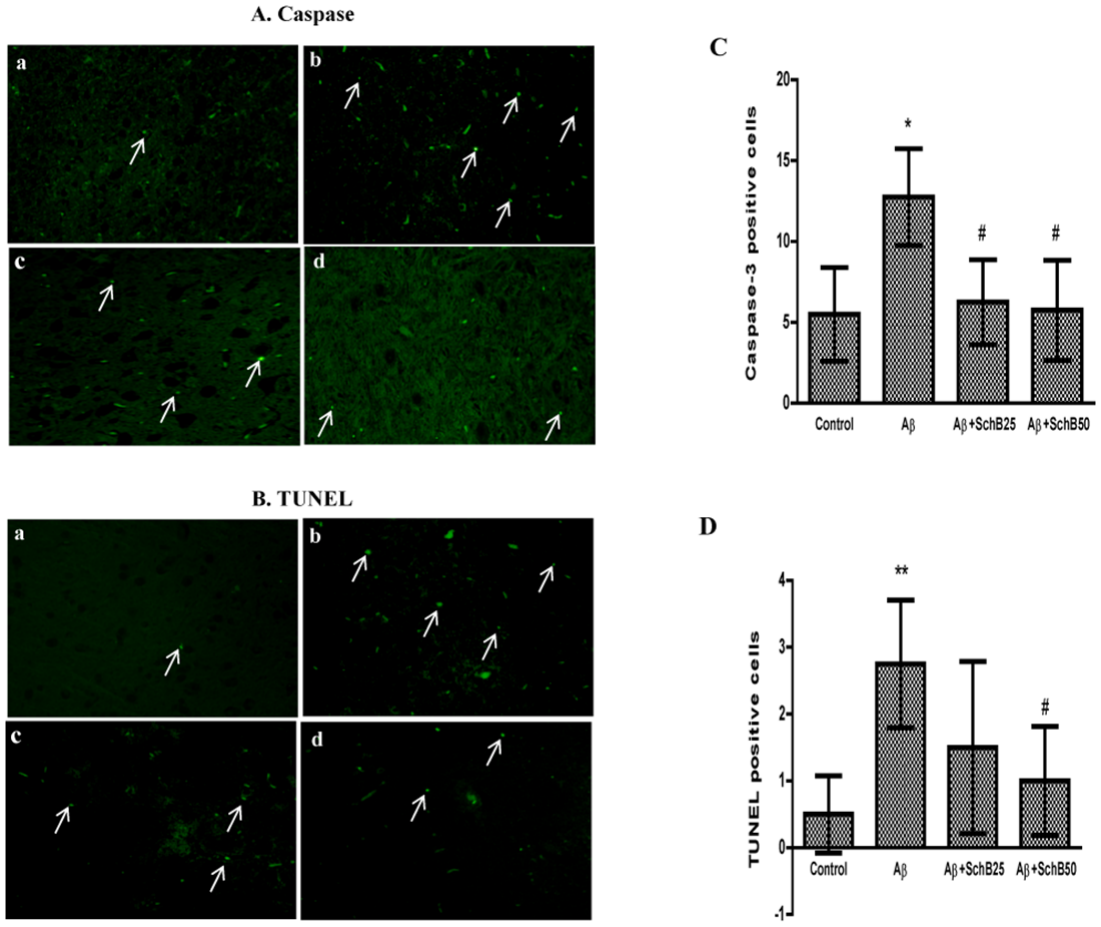
Attenuation of apoptotic markers levels by Sch B (25 and 50 mg/kg) treatment after Aβ/vehicle infusion. (a) Control (b) Aβ-infused (c) Aβ+SchB25 (d) Aβ+SchB50. Representative photomicrographs shows the immunofluorescence detection of (A) Caspase (B) TUNEL -positive cells. From each rat 5–6 coronal brain section were selected and about 15 fields of the cerebral cortex were analyzed. Scale bar = 5μm. The figures show quantification of (C) Caspase (D) TUNEL-positive cells. The results are given as the mean ± SD (n = 4). ** *P* < 0.01, * *P* < 0.05, compared with control group, and ^#^ *P* < 0.05, significantly different from Aβ-infused rats.

Neuronal cell death is a major causal factor in the development of AD [36], and oxidative stress plays critical role in the neuronal cell death. Thus, one of the mechanisms underlying the protective effect of Sch B on memory impairment is the inhibition of neuronal cell death as a consequence of suppression of oxidative damage in Aβ-infused rats. In order to assess the protective effect of Sch B on apoptotic neuronal cell death, cleaved caspase-3 expression was studied imunohistochemically. Aβ-infused rats showed a significant increase of cleaved caspase-3 expressed cells but the caspase-3 expression was effectively suppressed by the treatment with Sch B (Fig 6A and 6C). Likewise, TUNEL positive cells measured by histoimmunological method was markedly increased in Aβ infused rats but was dose-dependently reduced to 45.4% and 63.6% with 25 and 50 mg of Sch B, respectively (Fig 6B and 6D), indicating the inhibition of apoptotic cell death might be the major cause of the preventive effect of Sch B on neuronal cell death.

## Discussion

Since our previous study showed that Sch B dose-dependently improved scopolamine-induced amnesia in mice [15], the effect of Sch B was studied on Aβ model rats and showed Sch B was also effective in preventing the Aβ induced brain dysfunctions including memory deficit.

Aβ is thought to be the instigator of the memory deficit and dementia driving AD [42], and ICV infusion of Aβ in rats has been demonstrated as a successful method for establishing an AD animal model [9, 43]. Our present study demonstrated that Sch B appears to regulate a number of features defining AD, such as oxidative-nitrosative stress, glial cell activation, neuronal apoptosis, RAGE/NF-κB/MAPK activation, and autophagy inhibition in addition to cognitive impairment induced by Aβ infusion.

Given the proximal role and devastating effect that oxidative stress plays in AD pathogenesis [44], a therapeutic strategy based on fighting oxidative stress appears reasonable [45]. As such, increasing GSH level in the mitochondria might be an important therapeutic approach to prevent cell death in oxidative-stress-linked, age-dependent neurodegenerative disorders [46]. In this study, we demonstrated that the antioxidant lignan Sch B inhibited Aβ-induced oxidative injuries measured in terms of decreased GSH and SOD levels, with a parallel increase in MDA levels. Additionally, treatment with Sch B successfully decreased the superoxide production as shown in DHE staining and showed higher ORAC values. Altogether, these results support the antioxidant potential of Sch B might be a primary effect against Aβ infusion, as the brain is susceptible to oxidative stress compared with other tissues, there are various protective proteins to combat this stress such as HSP. In the present study, it was found the expression of the genes encoding HSPs was modulated by Sch B in neurons. HSPs serve as molecular chaperones, and among the various HSPs, HSP70 has recently been demonstrated to play a neuroprotective role against oxidative stress [47]. Although Sch B has been shown to enhance the HSP expression in cardiac [48] and hepatotoxicity injuries [49], this is the first study to report the HSP expression is involved in the protective function of Sch B against brain dysfunction.

Increasing body of immunohistological and molecular findings showing that inflammatory processes are pre-eminent and constant aspects of neuropathology generated by the Aβ toxicity, support the notion that the previously underappreciated glial activation plays a critical role in the pathogenesis of brain lesions subsequent to Aβ deposition [50]. Acute activation of glial cells may have important beneficial effects in the recovery of the central nervous system from a variety of insults; it is believed that the persistent activation amplifies inflammatory responses leading to worsening of the consequences of injury [51]. Although, we studied the role of Sch B against gliosis and astrocytosis markers (data not shown) in Aβ-infused rats, the results from the study need further confirmation. Consistently with these observations, it was found in the present study, many of the inflammatory markers TNF-α, IL-1β, IL-6, iNOS and COX-2 examined were significantly modulated in Sch B-treated rats. Although it is known that Sch B has anti-inflammatory activity such as reported that Sch B inhibits silica-induced inflammatory response through inhibition of TGF production and NF-κB activation [52, 53], the suppressive effect of Sch B on glial activation might be also involved in the mechanism of Sch B mediated inhibition of the inflammatory markers expression.

On the other hand, a growing body of evidence suggests that the interaction of AGEs with their cell surface receptor, RAGE, mediates NF-κB activation, which will increase the production of inflammatory markers and enhance the activation of both microglia cells and astrocytes, ultimately resulting in neurodegenerative disorders [54]. It has been hypothesized that, in pathological conditions such as AD, RAGE could act as a co-factor to facilitate the transport of Aβ across the blood-brain barrier and increase the cerebral Aβ levels, with consequent cellular dysfunction, induction of oxidative stress, loss of cellular vitality, and apoptosis [55]. Recent studies suggest that specific blocking of this receptor has potential as a future therapeutic approach to decrease the brain uptake of Aβ from blood [56]. In this study, it was demonstrated that, in addition to MAPK (p38, ERK) and NF-κB inhibition, Sch B significantly inhibited the RAGE expression. This suggests the potential role of Sch B in inactivation of the RAGE/NF-κB/MAPK axis.

In addition, RAGE is linked with other ligands, as AGE stimulates iNOS expression [57]. In the present study, Sch B significantly suppressed Aβ-induced nitrite production and iNOS, suggesting that the anti-inflammatory activity of Sch B was also expressed through the inhibition of NO release.

Autophagy is a self-cleaning cellular housekeeping mechanism that plays an important role in numerous pathologies [58]. A heterozygous deletion of the autophagy marker beclin-1 in Tg2576 mice increases intra-neuronal Aβ accumulation, extracellular Aβ deposition, and neurodegeneration [59], suggesting that autophagy plays a key protective role against AD. Recent findings further support the notion that modulation of autophagy, such as through induction of beclin 1, may represent a novel therapeutic strategy for AD. In the present study, treatment with Sch B moderately increased the expression of beclin-1, an autophagy marker. This observation together with HSP over expression by Sch B suggests the lower neuronal Aβ accumulation observed in Sch B treated rats was resulted from both the inhibition of uptake through RAGE and autophagic scavenging of accumulated Aβ. Our study for the first time identified the novel function of Sch B as an up-regulatory moderator of beclin-1 autophagy proteins even though the activity was not marked.

**Fig 7:**
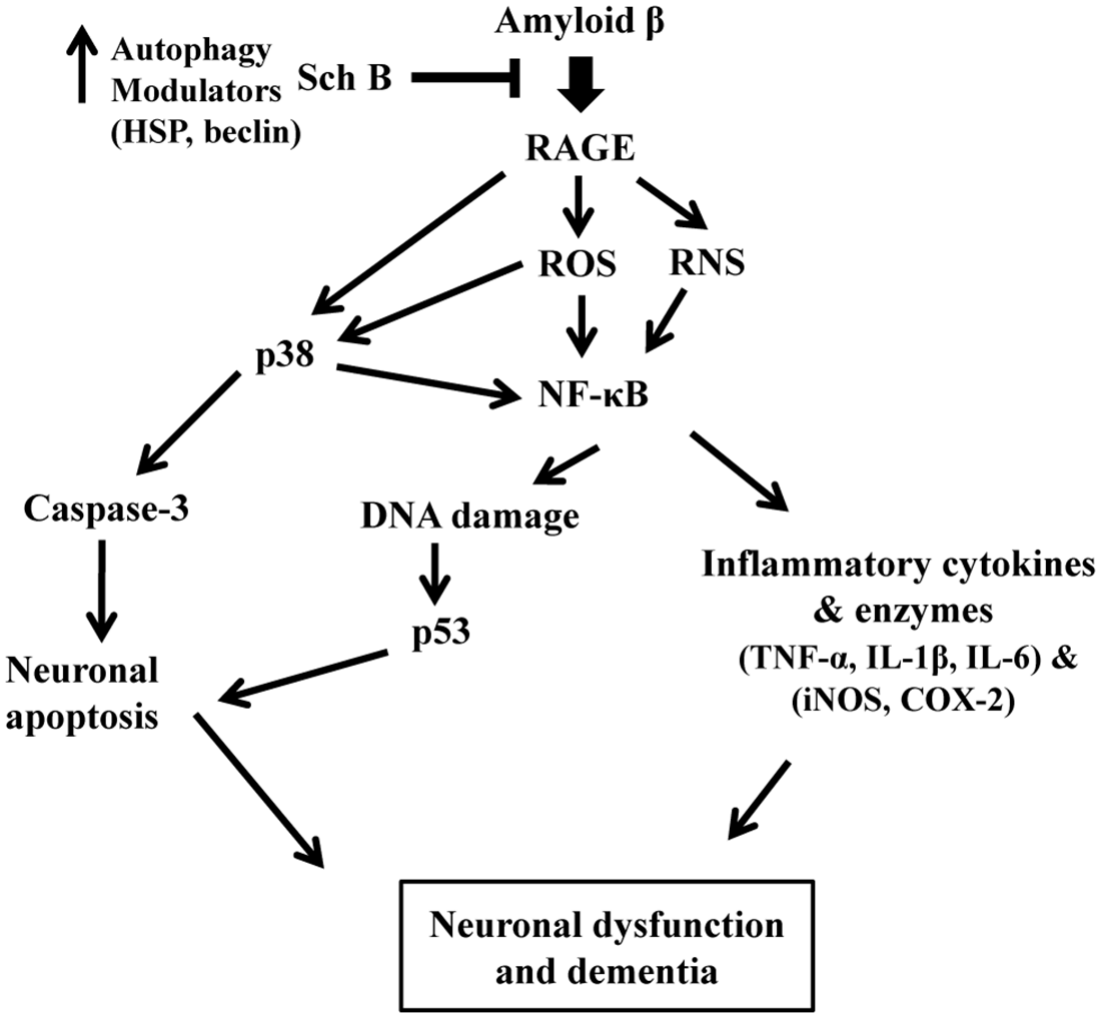
Schematic representation of SchB mechanism of action in preventing Aβ induced neurotoxicity. ROS; reactive oxygen species, RNS; reactive nitrogen species, RAGE; receptor for advanced glycation end products.

This research intended to determine the neuro-protective mechanism (Fig 7) of Sch B against Aβ-infused AD model rats, and revealed the potential cognitive improvement role of Sch B as an RAGE/NF-κB/MAPK axis inhibitor with antioxidant and anti-inflammatory activities. Finding of the role of Sch B as an autophagy promoter leads to develop effective protection strategies not only for amyloidosis but also for chronic conditions where autophagy is known to play a key protective role. Although additional confirmation is required, this study showed a novel mechanistic approach of Sch B as a potential agent for preventing or retarding the development and progression of AD.
